# Monosaccharide coating modulate the intracellular trafficking of gold nanoparticles in dendritic cells

**DOI:** 10.1016/j.mtbio.2024.101371

**Published:** 2024-11-27

**Authors:** Meshal A. Alobaid, Sarah-Jane Richards, Morgan R. Alexander, Matthew I. Gibson, Amir M. Ghaemmaghami

**Affiliations:** a Immunology & Immuno-bioengineering, School of Life Sciences, Faculty of Medicine and Health Sciences, University of Nottingham, Nottingham, NG7 2RD, United Kingdom; b Biology, Immunology, American International University, Al-Jahra, Saad Al Abdullah, Kuwait; c Warwick Medical School, Department of Chemistry, University of Warwick, Coventry, CV4 7AL, United Kingdom; d School of Pharmacy, University of Nottingham, United Kingdom; e Department of Chemistry, University of Manchester, Oxford Road, Manchester, M13 9PL, United Kingdom; f Manchester Institute of Biotechnology, University of Manchester, 131 Princess Street, Manchester, M1 7DN, United Kingdom

**Keywords:** Dendritic cells, T cells, Carbohydrates, Monosaccharides, Immune modulation, Gold nanoparticles, Surface coating

## Abstract

Dendritic cells (DCs) have emerged as a promising target for drug delivery and immune modulation due to their pivotal role in initiating the adaptive immune response. Gold nanoparticles (AuNPs) have garnered interest as a platform for targeted drug delivery due to their biocompatibility, low toxicity and precise control over size, morphology and surface functionalization. Our investigation aimed to elucidate the intracellular uptake and trafficking of AuNPs coated with different combinations of monosaccharides (mannose, galactose, and fucose) in DCs. We used 30 unique polymer-tethered monosaccharide combinations to coat 16 nm diameter spherical gold nanoparticles and investigated their effect on DCs phenotype, uptake, and intracellular trafficking.

DCs internalized AuNPs coated with 100 % fucose, 100 % mannose, 90 % mannose +10 % galactose, and 80 % mannose +20 % galactose with highest efficiency. Flow cytometry analysis indicated that 100 % fucose-coated AuNPs showed increased lysosomal and endosomal contents compared to other conditions and uncoated AuNPs. Imaging flow cytometry further demonstrated that 100 % fucose-coated AuNPs had enhanced co-localization with lysosomes, while 100 % mannose-coated AuNPs exhibited higher co-localization with endosomes.

Furthermore, our data showed that the uptake of carbohydrate-coated AuNPs predominantly occurred through receptor-mediated endocytosis, as evidenced by a marked reduction of uptake upon treatment of DCs with methyl-β-cyclodextrins, known to disrupt receptor-mediated endocytosis. These findings highlight the utility of carbohydrate coatings to enable more targeted delivery of nanoparticles and their payload to distinct intracellular compartments in immune cells with potential applications in drug delivery and immunotherapy.

## Graphical abstract

**Figure:**
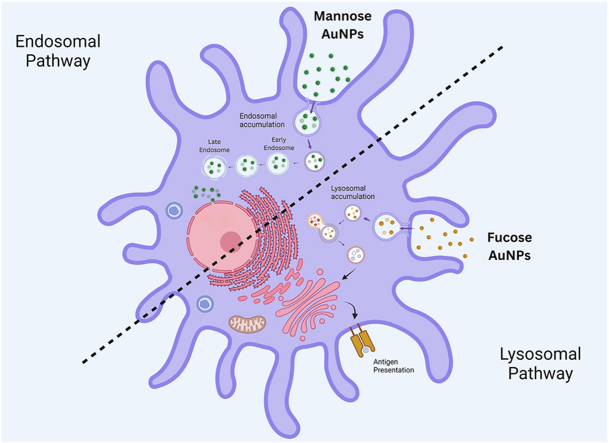

**Key findings**

• Fucose coated AuNP have greater uptake than Galactose and mannose coated AuNPs
• Fucose coated AuNP are found to largely localize in endosomes and not lysosomes
• Mannose coated AuNP are found to localize in lysosomes
• Uncoated AuNPs have shown higher uptake by DCs than sAuNPs but have low localization with lysosomes and endosomes
• Combination of galactose with mannose or fucose lowers the uptake of AuNPs

## 1 Introduction

Nanoparticles (NPs) have been extensively researched as a promising tool for drug delivery, vaccination, molecular nanoparticle imaging, and cancer therapy [1]. NPs are defined as ultrafine particles of diameters less than 100 nm [2]. NPs have gained attention for their properties attributed to the small size in many fields including cancer therapies and drug delivery [3]. Their minute size allows these particles to infiltrate different kind of tissues which is particularly important when considering payload distribution in solid tumours hence positioning NPs as ideal means for anti-cancer drug delivery [4]. Current technology advancements have facilitated synthesising NPs from different materials. Several studies have shown that NPs of different chemistries and sizes have demonstrated to have outcomes on the cellular level specifically on immune cells [5]. Gold is one of the materials that is used for NP fabrication because of its inert chemical properties as well as physical stability [6]. Gold nanoparticles (AuNPs) have been shown to have no toxic effects *in vivo* which in turn promoted more research to utilize them for drug delivery and other medical application [7].

Gold nanoparticles (AuNPs) have gained significant attention due to being highly suitable for biomedical applications. In cancer diagnostics, AuNPs have been widely explored as contrast agents for various imaging modalities, including computed tomography (CT), nuclear imaging, fluorescence, and photoacoustic imaging [8,9]. This versatility arises from their tunable optical properties, ease of functionalization, and biocompatibility. Beyond diagnostics, AuNPs also show great potential in therapeutic applications, particularly in photothermal therapy, where they absorb light and convert it into heat to selectively destroy cancer cells [10]. Furthermore, AuNPs are increasingly being utilized in targeted drug delivery systems, wherein they can improve the specificity and efficacy of chemotherapeutic agents, reducing side effects and enhancing tumor targeting through mechanisms such as receptor-mediated uptake [11]. Recent studies have demonstrated that AuNPs can be conjugated with dual-drug systems for more effective cancer treatment, highlighting their role in addressing challenges such as multidrug resistance and non-specific cytotoxicity [12]. These innovative uses of AuNPs in both diagnosis and treatment provide a promising platform for improving cancer therapy outcomes.

DCs are key modulators of the adaptive immune response. The migratory capacity of DCs makes them the ideal candidates for drug delivery and immune modulation [13]. AuNPs have been successfully used for targeting DCs. For example, it has been shown that AuNPs with polyelectrolyte multilayer coatings increased DCs ability to present antigens to CD8 T cells [14]. Another study showed that AuNPs coated with small interfering RNA can be successfully delivered to the nucleus through endosomal escape mechanisms utilizing pH and charge modifiers [15].

Modifying surface properties of AuNPs has been used as means for achieving intracellular concentration of the drugs or mediators in different cell compartments [16]. Recently, glycans gained attention in the field of drug delivery where they have been utilized to deliver payloads to DCs [17].

Glycans are abundantly expressed on cellular surfaces with high level of tissue and species specificity. Different pathologies could impact normal glycosylation patterns. For example, tumours are known to express aberrant glycosylation patterns which can be identified by the immune system [18]. C-type lectin receptors (CLRs) enable DCs to differentiate between self and foreign antigens through their monosaccharides and glycans. These CLRs are the one of the main mechanisms by which DCs differentiate foreign or aberrant antigens from self-antigens. Glycan coated nanoparticles have been shown to bind to CLRs and initiate an immune response. Studies have shown that DCs challenged with glycan functionalized nanoparticles coated with tumour antigen payloads are able to cross present the tumour antigens and activate CD8 T cells initiating a cytotoxic effect against tumours [19]. Sukyung Ahn et al. showed that AuNPs coated with tumour antigens were able to promote activation of CD8 T cells through DCs priming and activation [20].

Dextran is a member of the polysaccharide family that has been studied as potential coating for AuNPs for anti-tumour drug delivery directly to cancer cells [21]. Another study shows that AuNPs coated with Streptococcus glycoconjugates is a promising mean for vaccination through T-helper cell activation [22].

To achieve optimal drug delivery and desirable concentration, uptake and translocation of particles in the cellular compartment has been studied [23]. By understating particles rout of uptake whether it is phagocytosed passively “micropinocytosis” or actively such as clathrin or coveoli mediated, we can determine the fate of the payloads [24]. It has been shown that AuNPs can be distributed through tissues and into cells through passive uptake mostly because of their small size [25]. This is changed when the particles are coated with substances such as antibodies, antigens or other biological coatings to target specific cell populations [26]. The method of uptake is an important factor for the delivery of the payloads in targeted cells which is considered during vaccine design [27]. This can be achieved through targeting the active pathway with monosaccharide coatings ultimately leading to uptake of the particles.

Vaccination is reliant on antigen delivery to DCs in which the antigen is internalized, processed and presented to the T cells [28]. The phagosome containing the antigen matures and through trafficking it fuses with a lysosome and is degraded and presented in the context of MHCs molecules ready for presentations to T cells. This efficient antigen presentation process requires sufficient uptake of the antigen as well as rapid trafficking towards presentation of the antigen. Modulation of this step has been studied as means of delivery of drugs and small interfering RNA (siRNA) to the nuclease through prompting escape mechanisms from the phagosome compartment. This frees the payloads in the cell cytosol, such delivery mechanism can be used for siRNA in nanoparticle cancer therapy [29].

To identify optimal carbohydrate coating to achieve highest NP uptake by DCs we used a range of monosaccharides to coat polymer-coated AuNP (sAuNP) and investigated the impact of each coating on AuNP uptake and intracellular trafficking in DCs. Three different monosaccharides (mannose, galactose and fucose) were immobilized onto polymer tethers to create 30 unique combinations which were then immobilized onto 16 nm diameter spherical gold nanoparticles. The screening process involved assessing cellular uptake and impact on DC phenotype as well as localization of the sAuNPs into different intracellular compartments. Understanding such mechanisms allows the use of simple carbohydrates in targeted antigen delivery to DCs for the development vaccines including anti-tumour vaccines and gene therapies.

## 2 Methods

### 2.1 Synthesis and characterization of polymer-coated gold nanoparticles

Polymer coated gold nanoparticles were synthesized as we have provisory described [[30], [31], [32]]. Gold chloride trihydrate (HAuCl4; >49 % Au, ACS grade) were purchased from Sigma-Aldrich. Trisodium citrate (99.8 %) was purchased from Acros Organics. Ultra-high-quality water with a resistance of 18.2 MΩ × cm2 (at 25 °C) was obtained from a Millipore Milli-Q gradient machine fitted with a 0.22 μm filter. All other chemicals were used as received unless otherwise indicated.

### 2.2 Synthesis of citrate-stabilized gold nanoparticles

Briefly, 600 mL solution of a 0.85 mmol/L aqueous solution of HAuCl_4_ was heated to reflux in a scratch-free round bottomed flask. Then a ratio of 1:3 of Au/sodium citrate was made by adding 10.5 mL of a 0.5 mol/L of sodium citrate solution to the HAuCl_4_ in a single portion. The temperature was maintained at reflux for 30 min, during which time a deep-red coloration formed. The reaction mixture was then allowed to cool to room temperature over a period of 3 h. Assuming complete reduction of the HAuCl_4_ into the particles, the total gold concentration in the final solution was 0.17 mg/mL [32]. Particles were characterized by UV visible (UV–Vis) spectroscopy and TEM (supplementary Figure 1 and Fig. 2).

### 2.3 Physical and analytical methods

UV–Vis absorbance spectra were obtained on a Varian Cary 100 Bio spectrophotometer operating at 25 °C using 10 mm path length c-ettes with a total volume of 2.5 mL. The method of Fernig and coworkers was used to estimate the diameter of citrate-stabilized gold nanoparticle using UV–Vis spectroscopy [30].

Dynamic light scattering measurements were carried out on a Brookhaven Instruments system consisting of a BI-200SM goniometer and a BI-9000AT autocorrelator. A 100 mW Ar + ion laser (Lexel Lasers) operating at 488 nm was used. All measurements were performed at a temperature of 25.0 (±0.2) °C and at a scattering angle of 90°. Borosilicate cuvettes were used with a minimum of 3 mL of the nanoparticle solution. The nanoparticles were used at a concentration of ∼0.05 mg/mL (total gold mass) in Milli-Q water and passed through a 0.4 μm filter before measurement [32].

### 2.4 Gold nanoparticles monosaccharide coating

**Fig. 1:**
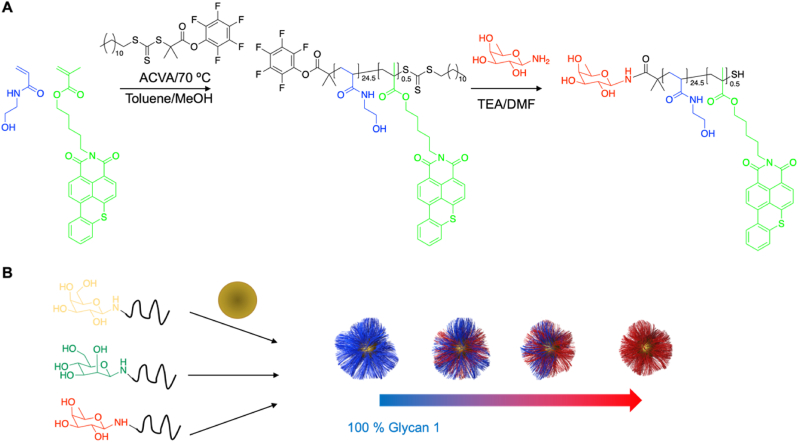
Schematic of the functionalization of gold nanoparticles with glycan-functionalized fluorescent polymers. A) co-polymerization of *N*-hydroxyethyl acrylamine and hostasol methacrylate using pentafluoropenyl (PFP) RAFT agent and the subsequent substitution of the PFP end group with an amino monosaccharide (galactosamine, mannosamine and fucosamine) B) Anchoring of glycopolymers on pre-formed 16 nm gold nanoparticles. (For interpretation of the references to color in this figure legend, the reader is referred to the Web version of this article.)

Poly(PHEA-co-hostasol methacrylate) polymer was synthesized as previously described by Gibson et al. [33] with a PFP end group for facile end modification with an amino-monosaccharide as previously outlined [31,34,35]. A stock of 10 mg/mL of the each of the polymers in high-purity water was made. The polymers were mixed in the in the desired to a volume of 100 μL (0.1 mg/mL polymer). This was added to 10 mL of the citrate-stabilized gold nanoparticle solution and agitated overnight in the absence of light on a roller. To remove excess unbound polymer, the particles were centrifuged for 30 min at 6010g. Following careful decantation of the supernatant, the particles were then redispersed in 10 mL of high-quality water, and the centrifugation−resuspension process was repeated for a total of five cycles. Complete removal of any unbound polymer was determined by fluorescence measurements on the supernatant (Supplementary Fig. 3) and the resulting AuNPs were gave fluorescence responses, where unfunctionalized AuNPs and PBS alone did not (Supplementary Fig. 4). Concentration was determined by UV–Vis, compared to before coating. The resulting polymers were found to be stable by UV–Vis (Supplementary Figs. 4 and 5) in PBS. After the final cycle, the particles were dispersed in 10 mL of high-quality water for future use. Fig. 1 provides schematic representation of this process.

### 2.5 Generation of human DCs from peripheral blood monocytes

Buffy coats from healthy individuals were obtained from National blood bank (Sheffield, U.K.) obtaining informed written consent and following approval from local ethics committee (Faculty of Medicine and Health Sciences Research Ethics Committee, University of Nottingham). Peripheral blood monocytes (PBMCs) separation was done by means of gradient centrifugation using Histopaque (Sigma-Aldrich, UK). To obtain CD14 monocytes, positive selection with magnetic beads using the cell separation system (Miltenyi Biotech, UK) was performed. Purity of cell separation was confirmed by flow cytometry which was typically >95 %. CD14 monocytes were cultured in 24 well tissue culture plates (1x10^6^ cell/well) at 37° in a 5 % CO_2_ humidified incubator for 6 days in complete RPMI media containing 10 % heat inactivated Foetal Bovine Serum (FBS), 100 mg/mL streptomycin, 100 U/mL penicillin and 2 mM L-glutamine (all from Sigma-Aldrich) in the presence of 50 ng/mL GM-CSF and 250 U/mL IL-4 (both from Miltenyi biotech). On day 3 cells received fresh supplemented media [36,37].

### 2.6 Preparation of AuNPs and cell culture

Gold nanoparticles were centrifuged at 6010g 30 min. Excess water was aspirated, and the pellet was resuspended in RPMI 1640 supplemented with 10%FBS, 100 μg/mL streptomycin, 100 U/mL penicillin and 2 mM L-glutamine (all from Sigma-Aldrich). 100 μL of each of the resuspended gold particles are added to 400 μL of supplemented medium containing 300k dendritic cells. Incubation is carried out in 37 °C in a 5 % CO_2_ humidified incubator overnight where they are analyzed the following day.

### 2.7 Analytical flowcytometry

Cells were harvested and washed with PBA (PBS with 0.5 % bovine serum albumin and 0.1 % sodium azide). Incubation with labelled antibodies (MHC2 APC, MHC1 PE, CD86 FIT-C) (Miltenyi biotech) was then carried out in the dark at 4 °C for 20 min. Cells were then washed in PBA and fixed with 4 % paraformaldehyde until analysis. All samples are analyzed using FC500 Flow cytometer (Beckman Coulter) and data analysis was carried out using Weasel software.

### 2.8 Fixable viability LIVE/DEAD assay

Viability analysis was done using the LIVE/DEAD Fixable viability Cell Stain Kit (Thermo Fisher Scientific) following manufacturers protocol. Cells were washed with PBS, stained with 1 mL of LIVE/DEAD viability stain (Annexin V FIT-C), and incubated for 20 min in the dark at room temperature before fixing with 4 % formaldehyde solution. Flow cytometry was carried out on the FC500 (Beckman Coulter) [37,38].

### 2.9 Membrane cholesterol depletion

Dendritic cells were treated with 10 mM of Methyl-β-cyclodextrin suspended in complete RPMI 1640 medium for 60 min in an incubator. Following incubation, the cells are centrifuged and medium discarded and resuspended in new supplemented complete medium where they are ready for experimentation [3,39].

### 2.10 Image flowcytometry

Dendritic cells conditioned with sAuNP or controls are stained intracellularly for the assessment of particle uptake, lysosomes, endosomes, and nucleus. Cells are fixed for 10 min using 5 % paraformaldehyde and then resuspended and washed with 0.5 saponin (Biolegend) twice before staining. Cells are incubated with fluorochrome conjugated monoclonal antibodies CD107a-PE-Vio615 (LAMP-1), CD71-PE-vio770 (Endosomes) (both from Biolegend) and DAPI (Sigma Aldrich) for 30 min. AuNP conjugated with Hostasol green was used to look at uptake. After incubation cells are washed with 0.5 saponin, resuspended and fixed with 5 % paraformaldehyde until analysis. Samples are analyzed using the MKII imagestream by Amnis and data is analyzed using IDEAS v6.2 Amins software.

### 2.11 Statistical analysis

Statistical significance of the Data was obtained using GraphPad Prism 7 (GraphPad Software, San Diego, CA). Therefore, paired t tests or ordinary one-way ANOVA was used when comparing between two groups. Statistically significant p values were considered to be equal or lower than 0.05.

## 3 Results

### 3.1 sAuNPs do not activate immature dendritic cells

**Fig. 2:**
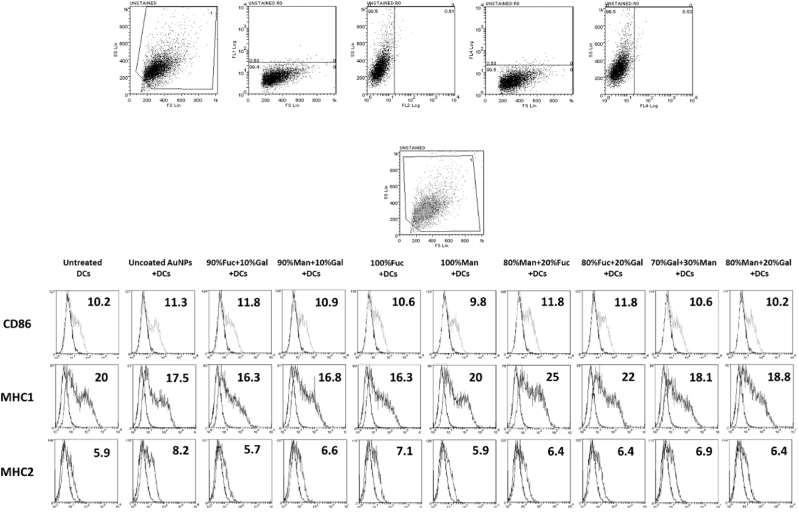
Flowcytometry phenotypic analysis and gating strategy of costimulatory markers CD86, MHC1 and MHC2 for sAuNP conditioned DCs. Data show no change in the costimulatory markers suggesting no stimulatory effects of the sAuNP on DCs. Data shown is representative of 3 donors.

Following confirmation of DC differentiation from monocytes (Supplementary Fig. 6), we examined the potential of sAuNPs to induce maturation in immature dendritic cells (iDCs) by assessing changes in surface phenotype and IDO enzyme activity, in comparison to uncoated AuNPs. Specifically, CD86, MHC1 (HLA-ABC) and MHC2 (HLA-DR) surface markers were analyzed, and IDO activity was measured. Fig. 2 shows that there were no notable differences in CD86, MHC1 or MHC2 expression in DCs treated with sAuNPs, compared to those treated with uncoated AuNPs. Similarly, Supplementary Fig. 7 shows that there was no increase in IDO activity in sAuNP-treated cells, indicating that the particles did not activate DCs. Collectively, these findings suggest that the AuNPs and sAuNPs used in this study (Table 1) did not promote DC maturation.

**Table 1:** Monosaccharide combinations used to coat AuNPs.

sAuNP monosaccharide combinations		
100%Mannose	100%Galactose	100%Fucose
90%Mannose 10%Galactose	90%Galactose 10%Fucose	90%Fucose 10%Mannose
80%Mannose 20%Galactose	80%Galactose 20%Fucose	80%Fucose 20%Mannose
70%Mannose 30%Galactose	70%Galactose 30%Fucose	70%Fucose 30%Mannose
60%Mannose 40%Galactose	60%Galactose 40%Fucose	60%Fucose 40%Mannose
50%Mannose 50%Galactose	50%Galactose 50%Fucose	50%Fucose 50%Mannose
40%Mannose 60%Galactose	40%Galactose 60%Fucose	40%Fucose 60%Mannose
30%Mannose 70%Galactose	30%Galactose 70%Fucose	30%Fucose 70%Mannose
20%Mannose 80%Galactose	20%Galactose 80%Fucose	20%Fucose 80%Mannose
10%Mannose 90%Galactose	10%Galactose 90%Fucose	10%Fucose 90%Mannose

### 3.2 Screening of sAuNPs uptake by dendritic cells

**Fig. 3:**
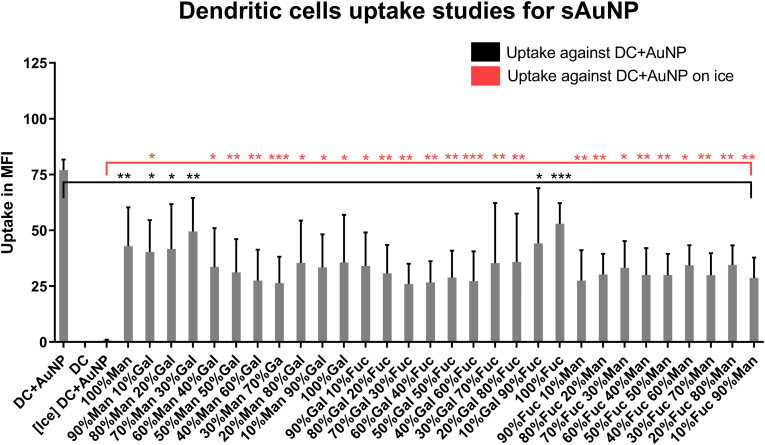
Flowcytometry data show uptake studies for sAuNP conditioned DCs. Different combinations have shown to promote uptake more than others where increase uptake is seen in uncoated AuNPs, 100%mannose, 90%mannose+10%galactose, 80%mannose+20%galactose, 70%mannose+30%galactose, 60%mannose+40%galactose, 100%fucose, 90%fucose+10%galactose. AuNP uptake by DCs is subtantially abroagted at 4 °C (incubated on ice). Data show are MFI + -SD of >3 separate donors. Data shown are a mean ± SD of three independent donors where ∗p < 0.05, ∗∗p < 0.01, ∗∗∗p < 0.001, ∗∗∗∗p < 0.0001.

To evaluate the cellular uptake of AuNPs and sAuNPs, we utilized conjugated particles with the fluorophore Hostasol Green. Hostasol Green fluorescence was measured by flow cytometry to quantify particle uptake. Untreated iDCs and cells incubated at 4 °C before particle addition were included as controls. As shown in Fig. 3, uptake of sAuNPs with different monosaccharide coatings, as well as uncoated particles, was examined. The results indicate that certain monosaccharide combinations promote the uptake of AuNPs more than others. Notably, the highest uptake was observed in uncoated AuNPs. Based on the uptake patterns shown in Table 2, sAuNPs with the highest and lowest uptake were selected for additional experiments to further investigate these patterns.

**Table 2:** sAuNP conditions chosen to take forward into experiments depending on highest and lowest uptake.

Highest uptake	Lowest uptake
100%Mannose	80%Mannose 20%Fucose
90%Mannose 10%Galactose	20%Mannose 80%Fucose
80%Mannose 30%Galactose	60%Galactose 40%Fucose
70%Mannose 30%Galactose	40%Galactose 60%Fucose
60%Mannose 40%Galactose	30%Galactose 70%Fucose
100%Fucose	20%Galactose 80%Fucose
90 % Fucose 10%Galactose	

### 3.3 sAuNP with high uptake are mainly through raft-mediated endocytosis

**Fig. 4:**
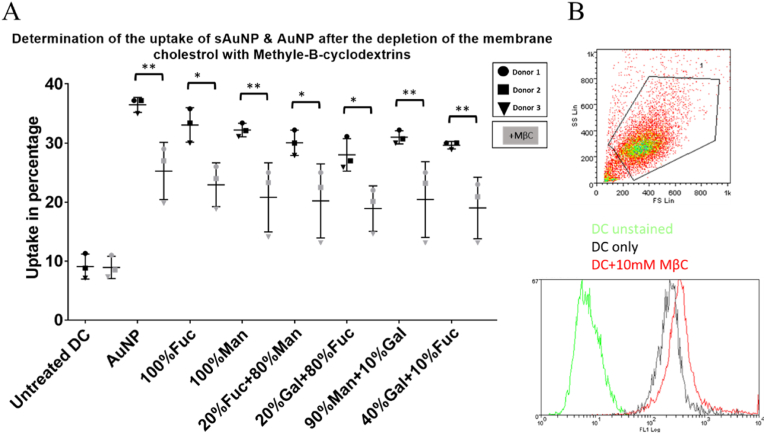
Uptake data obtained after the depletion of cholesterol on DC membrane using Methyl-β-cyclodextrins. A) There is a significant decrease in uptake of both coated and uncoated particles. This suggests that an active endocytosis is involved in the uptake of these particles however passive uptake is still involved in particle uptake B) DCs viability using Live/dead flowcytometry stain suggest that the treatment with 10 mM of Methyl-β-cyclodextrins did not affect DC viability. Data shown are a mean ± SD of three independent donors where ∗p < 0.05, ∗∗p < 0.01, ∗∗∗p < 0.001, ∗∗∗∗p < 0.0001.

In this study, we aimed to distinguish between passive (micropinocytosis) and active modes of sAuNPs uptake to better understand the intracellular trafficking of these particles. To accomplish this, we utilized methyl-β-cyclodextrins (MβC), a well-known cholesterol-depleting agent that disrupts cholesterol-rich lipid rafts with knock-on effect on receptor-mediated endocytosis, leaving passive micropinocytosis broadly intact [39,40]. We found that treatment with MβC resulted in a significant decrease in uptake of different sAuNPs, as demonstrated in Fig. 4a. Importantly, this effect was not due to any adverse impact on cell viability, as evidenced by the live/dead assay results shown in Fig. 4b. These findings support the notion that receptor-mediated endocytosis is the predominant mode of sAuNPs internalization most likely through c-type lectins (e.g. DC-SIGN and mannose receptor) expressed by DCs [37,39].

### 3.4 sAuNP modulates lysosomal and endosomal content in dendritic cells

**Fig. 5:**
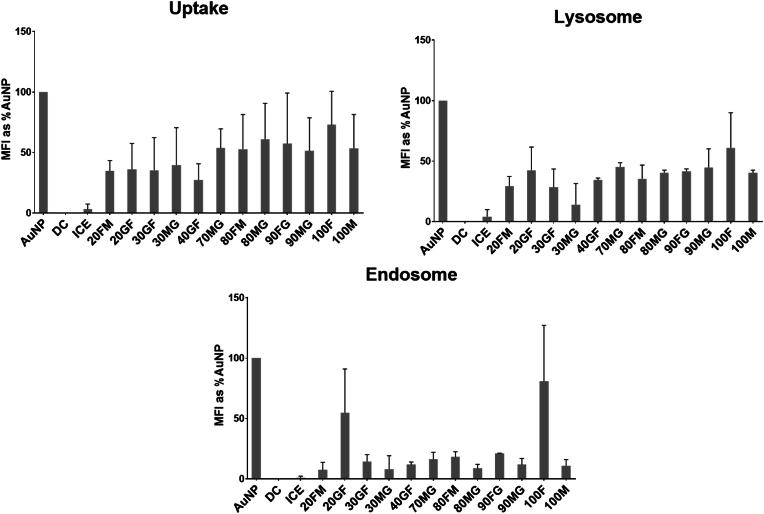
Flowcytometric analysis for uptake, lysosomal and endosomal contents for sAuNP conditioned DCs after 24 h incubation. High lysosomal content is seen in 100%fucose, 80%fucose+20%galactose and 100%mannose. These conditions also expressed high endosomal content except for 100%mannose. Data shown as mean ± SD for 3 donors.

In order to gain deeper insights into the cellular uptake of sAuNPs and the potential effects of various carbohydrate coatings on their intracellular trafficking, we examined the lysosomal and endosomal content in DCs exposed to different conditions. The experiments were carried out at three time points (1, 6, and 24 h) to evaluate the uptake of particles in relation to the lysosomal and endosomal content of cells. Supplementary Fig. 8 shows that the uptake of sAuNPs increased over time, reaching its peak after 24 h. The lysosomal and endosomal content remained relatively stable during the first 6 h and only increased significantly after 24 h. Fig. 5, which depicts the 24-h time point, reveals that 100 % fucose, 80 % fucose +20 % galactose, and 100 % mannose coatings resulted in higher lysosomal content in iDCs treated with sAuNPs. Additionally, these conditions led to high endosomal content, with the exception of 100 % mannose. There was no impact on DC viability following sAuNPs treatment (Supplementary Fig. 9).

### 3.5 Fucose and mannose sAuNPs promote particle trafficking at lysosomes and endosomes respectively in dendritic cells

**Fig. 6:**
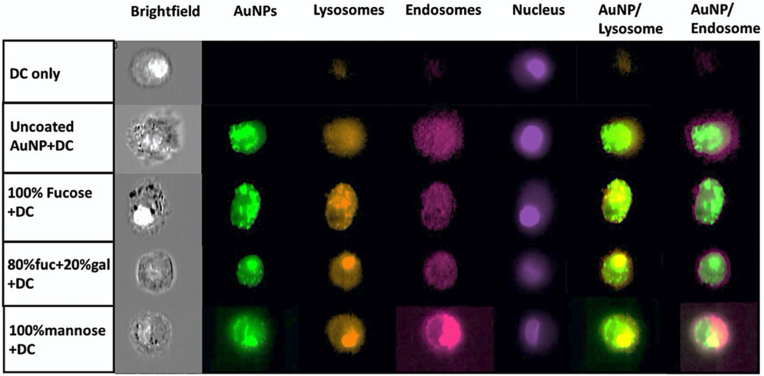
Images representing sAuNPs uptake Lysosomes (orange), endosomes (pink) and nucleus (purple) in DCs. Data confirms uptake by the conditions where most uptakes are seen in 100 % fucose. It is also where most lysosomal co-localization can be noted followed by 80%fuc+20%gal. These conditions show reduced endosomal co-localization suggesting rapid particle delivery to lysosomal contents. On the other hand. 100%Mannose show increased endosomal co-localization suggested delayed delivery of the particles to endosomes. Uncoated AuNPs show increased uptake but reduced co-localization for both endosomal and lysosomal contents. Data shown are representative images of 60x of 3 donors with 1000 images analyzed per donor. (For interpretation of the references to color in this figure legend, the reader is referred to the Web version of this article.)

**Fig. 7:**
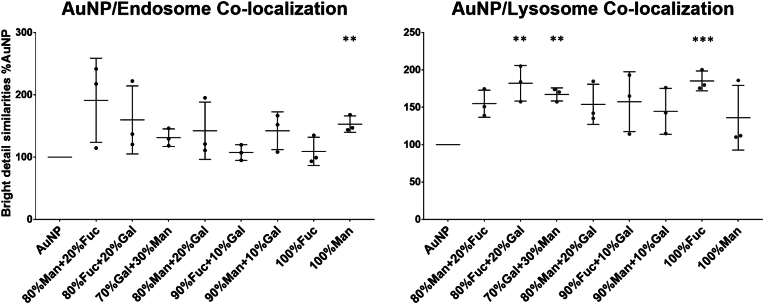
Illustrating Gold particle co-localization studies in lysosomal and endosomal contents in conditioned DCs. Data show that 100 % fucose, 80%fucose+20%galactose and 70%gal+30%man have significant increase in sAuNPs/lysosomal co-localization compared to uncoated AuNPs. These conditions show no significant increase in the sAuNPs/endosomal co-localization. On the other hand, 100%mannose show a significant increase in sAuNPs/endosomal co-localization while no significant increase in the sAuNPs/lysosomal co-localization compared to uncoated AuNPs. Data shown are mean ± SD of three independent donors where ∗p < 0.05, ∗∗p < 0.01, ∗∗∗p < 0.001, ∗∗∗∗p < 0.0001.

To investigate the relationship between rapid uptake of sAuNPs and their potential role in antigen presentation through lysosomal and endosomal compartments, we analyzed the localization of sAuNPs in these compartments. Our objective was to identify sAuNPs that are efficiently internalized and trafficked for antigenic payload degradation via lysosomal contents, leading to efficient antigen presentation. Fig. 6, Fig. 7indicate that sAuNPs with coatings of 100 % fucose, 80 % fucose+20 % galactose, and 70 % galactose+30 % mannose exhibit significant co-localization with sAuNPs and lysosomes compared to uncoated AuNPs. However, these coatings show no significant increase in the sAuNPs/endosomal co-localization. In contrast, sAuNPs with 100 % mannose coating exhibit a significant increase in sAuNPs/endosomal co-localization but no significant increase in the sAuNPs/lysosomal co-localization compared to uncoated AuNPs. Supplementary Table 1 shows the data for each donor used to quantify co-localization.

## 4 Discussions

Metal nanoparticles such as AuNps have emerged as promising nanosystems for various therapeutic and diagnostic approaches. Size, shape and surface properties of NPs can be modified to enhance their targeting and accumulation. There are various examples of using synthetic or natural polymers and ligands as costings to improve NPs uptake and delivery which are reviewed in detail elsewhere [41,42]. Glycosylated, polymer stabilized, AuNPs have been investigated as a promising platform for drug delivery and targeting immune cells including DCs. The optimization of AuNP coatings enables targeted uptake of these particles and enhances uptake through different coatings. Using AuNPs to deliver tumor antigens to DCs for anti-tumor vaccination has shown great potential [43,44]. Coating AuNPs with single or combination of different monosaccharides could potentially change the fate (e.g. uptake efficiency, intracellular trafficking) of AuNPs due to preferential interaction with different C-type lectin receptors (CLRs) on DCs surface

The size and shape of nanoparticles are critical factors influencing their biological interactions, including their cellular internalization, biodistribution, and intracellular trafficking. Various studies have demonstrated that nanoparticles in the size range of 10–50 nm exhibit optimal cellular uptake due to their ability to engage more effectively with cell surface receptors and avoid rapid clearance by the reticuloendothelial system [45,46]. Spherical nanoparticles are particularly advantageous as they tend to demonstrate higher internalization rates compared to rod-shaped or star-shaped nanoparticles, which are often less efficient due to their increased surface area and interaction with the extracellular matrix.

Studies have also shown that particles smaller than 10 nm tend to be rapidly cleared by renal filtration, whereas larger particles (above 50 nm) are more likely to be trapped by the liver and spleen [47]. Nanoparticles with sizes smaller than 10 nm–20 nm are generally internalized more efficiently through receptor-mediated endocytosis, often utilizing pathways such as clathrin-mediated or caveolin-mediated endocytosis, which are crucial for targeted delivery within cellular compartments [48]. This makes the 16 nm size an optimal balance, allowing sufficient circulation time to reach target cells while still maintaining efficient cellular uptake.

Furthermore, the spherical geometry of gold nanoparticles has been found to enhance their interaction with cellular membranes and enable more uniform distribution within the cell. Non-spherical particles such as rods or stars may aggregate or become less efficiently internalized, leading to uneven intracellular distribution and reduced therapeutic effectiveness [49]. Spherical nanoparticles are also more likely to remain stable in suspension, reducing the risk of agglomeration, which can compromise their functionality and therapeutic potential.

Therefore, the choice of 10 nm–20 nm (16 nm) spherical AuNPs was made based on these established advantages in size-dependent cellular uptake, biodistribution, and intracellular stability, making them ideal candidates for investigating intracellular trafficking and optimizing targeted drug delivery strategies in dendritic cells.

Different monosaccharides interact with different surface CLRs, and a combination of monosaccharides could enhance recognition and uptake of a specific particle. Monosaccharide coatings and in particular their combinatorial use have not been extensively studied in the context of targeted drug delivery to immune cells and vaccination. In this study, we investigated how different monosaccharide combinations modulate the uptake of AuNPs by DCs and their intra cellular trafficking once taken up. Our findings provide information on the monosaccharide combinations that exhibit the highest uptake and rapid AuNPs localization towards lysosomal compartments. These findings identify ideal coatings for efficient antigen delivery to DCs with important implications for designing new vaccine delivery formulations.

Li et al. demonstrated the critical role of nanoparticle size and surface modification in determining the pathways of cellular uptake and intracellular processing [50]. Their findings indicate that surface-functionalized AuNPs can preferentially enter cells through clathrin-mediated endocytosis, which plays a central role in ensuring that nanoparticles avoid lysosomal degradation and instead traffic to compartments such as the endoplasmic reticulum. These results align with our study, where 16 nm spherical AuNPs were efficiently internalized and trafficked to specific intracellular compartments, facilitating their intended function in antigen processing.

Ho et al. further elucidated how surface chemistry affects intracellular trafficking, showing that PEGylated AuNPs enhance cellular uptake while avoiding rapid sequestration in lysosomes [51]. This supports our findings that nanoparticle surface modifications are crucial for optimizing intracellular delivery and prolonging retention in key cellular compartments. Additionally, Liu et al. explored how AuNP size and surface charge influence not only receptor-mediated endocytosis but also downstream intracellular fate [52]. They demonstrated that nanoparticles with optimal sizes, such as the 16 nm spherical AuNPs used in our study, are more likely to escape from endosomes and enter the cytoplasm, thereby improving their efficiency in drug delivery and antigen presentation.

Initially we investigated the impact of various combinations of sAuNPs on DCs, with a focus on changes in cellular phenotype and IDO enzymatic activity. The expressions of CD86, MHC1, and MHC2 are commonly used markers for DC activation and maturation. However, our results showed no significant changes in DC maturation in response to different sAuNP combinations, indicating no stimulatory effects by sAuNPs on DCs. Furthermore, analysis of IDO enzyme activity showed no significant changes in these cells. It is worth noting that previous studies on the effects of AuNPs on DCs have reported conflicting results, with some studies reporting stimulation and maturation of DCs while others showing opposing findings. These discrepancies may be attributed to the combination of surface charge and size of particles, which can affect their ability to cluster and bind to CLRs and activate DCs [53,54].

The use of monosaccharides as coatings on AuNPs has been shown to enhance uptake and potentially target delivery to different cellular compartments. While mannose is a well-known ligand for DC-SIGN and MR, and has been widely used as a coating for nanoparticles, the data on the use of other monosaccharides for nanoparticle coating, and in particular their combinatorial use is scarce [55]. In our experiments, we examined the effect of different combinations of monosaccharides on AuNP uptake by DCs. Our data showed that different monosaccharide combinations had varying effects on uptake, with some enhancing uptake while others reducing it compared to uncoated AuNPs.

Combinations consisting of mannose and galactose showed a direct correlation between uptake and the content of mannose, with greater uptake observed in combinations with higher mannose content. This is not surprising, as most intracellular pathogens penetrate cells through binding to CLRs using mannose as a ligand. Similarly, fucose showed a proportional increase in uptake with increasing fucose content. However, the incorporation of galactose into combinations of fucose or mannose decreased uptake into cells, likely due to the fact that the CLRs responsible for fucose and mannose uptake (MR and DC-SIGN) are much more highly expressed in DCs than the CLR responsible for galactose uptake (MGL) [56]. Interestingly, combinations consisting of both fucose and mannose showed low uptake and internalization by DCs. This suggests a rate limiting uptake of sAuNPs when DCs are presented with both monosaccharides simultaneously potentially due to competitive inhibition or receptor saturation, where the presence of both ligands simultaneously interferes with receptor engagement. This is in contrast to studies showing efficient internalization of *Faciola hepatica* antigens that contain both mannose and fucose moieties [57]. However, this is likely due to different dynamics governing uptake of soluble antigens versus particulated ones.

**Fig. 8:**
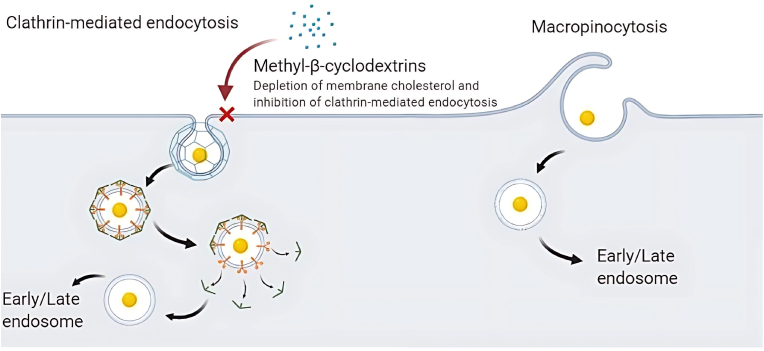
Depletion of cholesterol on DC membrane using Methyl-β-cyclodextrins. This enables the inhibition of the formation of lipid rafts and there for disabling active endocytosis but not passive endocytosis. This allows the understating of the means in which particles are internalized differentiating between active and passive endocytosis.

**Fig. 9:**
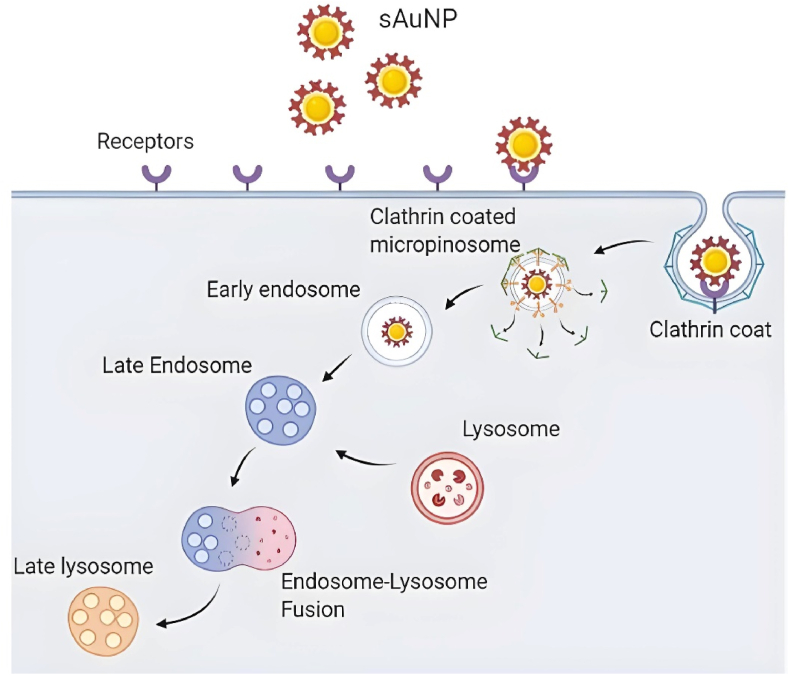
Illustrates the mechanism of sAuNP uptake by dendritic cells (DCs) through active endocytosis. Upon internalization, the particles are encapsulated by a protein coat, resulting in invagination of the plasma membrane and the formation of an early endosome. Subsequently, the early endosome matures into a late endosome, which ultimately fuses with a lysosome. Within the lysosome, the enzymatic activity degrades the contents of sAuNP, which then proceed for antigen presentation on dendritic cells.

To better understand the observed sAuNP uptake patterns, we investigated the effects of active and passive endocytosis on both AuNPs and sAuNPs. We employed MβC to deplete cholesterol from the lipid bilayer, thereby reducing lipid raft formation and active endocytosis while leaving passive endocytosis (micropinocytosis) unaffected [58]. This allowed us to gain insights into the primary pathways involved in the uptake of these particles. Our data indicate a decrease in the uptake of both sAuNPs and AuNPs in MβC-treated cells compared to untreated cells, suggesting that both monosaccharide-coated and uncoated AuNPs are taken up via receptor mediated pathways. As we previously stated, AuNPs can aggregate through surface charges and engage CLRs leading to their internalization. It is unsurprising that monosaccharides on sAuNPs interact with CLRs, which play a significant role in internalization. However, our findings also demonstrate that small particles can gain access to cells through passive micropinocytosis without interacting with surface CLRs (See Fig. 8, Fig. 9).

We identified high and low uptake conditions and investigated the fate of sAuNPs by studying the endosomal and lysosomal content. The selected conditions with the highest uptake (100%mannose, 90%mannose+10%galactose, 80%mannose+20%galactose, 70%mannose+30%galactose, 60%mannose+40%galactose, 100%fucose, 90%fucose+10%galactose) and lowest uptake (40%mannose+60%galactose, 40%galactose+60%fucose, 30%galactose+70%fucose, 20%galactose+80%fucose, 80%fucose+20%mannose, 80%mannose+20%fucose) revealed that active uptake leads to trafficking towards degradation through the lysosomal pathway, resulting in the presentation of antigens on MHC molecules. Flow cytometry analysis of the lysosomal and endosomal content for these conditions showed that the highest contents were for uncoated AuNPs, followed by 100%fucose. Additionally, 80%fucose+20%galactose coated AuNPs had an increase in endosomal content with no increase in lysosomal content compared to other sAuNPs. We further analyzed images of DC to determine the co-localization of particles in the endosomal or lysosomal contents. Our data show that, despite the increase in lysosomal and endosomal content of uncoated AuNPs, there is much less co-localization of these particles with both endosomal and lysosomal contents. AuNPs coated 100%fucose or 80%fucose+20%galactose exhibited a significant increase in co-localization of particles with the lysosomal content compared to uncoated AuNPs. In contrast, 100%mannose showed an increase in co-localization of particles with endosomal content. This indicates that fucose coatings enhance uptake and rapid delivery of payloads for presentation through MHC complex, while mannose is more suitable for coatings to escape lysosomal coatings and carry payloads directed for the nucleus for mRNA silencing.

## 5 Conclusions

In this study, we have demonstrated the ability of monosaccharide coatings to modulate -the uptake of payloads by dendritic cells (DCs). Our results indicate that specific monosaccharide coatings facilitate the rapid delivery of payloads, followed by lysosomal degradation. In particular, we have found that increasing the fucose content of the coating promotes fast payload delivery, while an increase in mannose content facilitates the retention of particles in the endosomal phase.

Our findings have important implications for the use of monosaccharide coatings in rapid antigen delivery and presentation in DCs, particularly in the context of anti-tumor therapies. Specifically, fucose-containing coatings may be particularly effective in enhancing DC-mediated antigen presentation in the fight against tumours. Similarly, the use of mannose-containing coatings may be particularly useful in therapies that require reduced endosomal escape of payloads and delivery into the inner cellular compartments, such as mRNA silencing therapies.

Our study provides valuable insights into the potential applications of monosaccharide coatings in the development of novel therapies that target DCs, and highlights the importance of further investigation into the use of these coatings for the treatment of various diseases.

## CRediT authorship contribution statement

**Meshal A. Alobaid:** Writing – original draft, Validation, Formal analysis, Data curation. **Sarah-Jane Richards:** Writing – review & editing, Writing – original draft, Resources, Methodology. **Morgan R. Alexander:** Writing – review & editing, Supervision, Funding acquisition. **Matthew I. Gibson:** Writing – review & editing, Supervision, Resources, Methodology, Funding acquisition. **Amir M. Ghaemmaghami:** Writing – review & editing, Writing – original draft, Supervision, Funding acquisition, Conceptualization.

## Declaration of competing interest

The authors declare the following financial interests/personal relationships which may be considered as potential competing interests:Amir Ghaemmaghami and Morgan Alexander report financial support, administrative support, and travel were provided by the United Kingdom 10.13039/501100000266Engineering and Physical Sciences Research Council. Matthew I. Gibson reports financial support was provided by 10.13039/501100000781European Research Council. Nothing to declare If there are other authors, they declare that they have no known competing financial interests or personal relationships that could have appeared to influence the work reported in this paper.

## Data Availability

Data will be made available on request.
