# Supplementary material for: Monosaccharide coating modulate the intracellular trafficking of gold nanoparticles in dendritic cells
Source: Mater Today Bio. 2024 Nov 27;29:101371. doi: 10.1016/j.mtbio.2024.101371 (PMC11652954; doi:10.1016/j.mtbio.2024.101371)
Supplement: Multimedia component 1 [file mmc1.docx]

**Supplementary information**


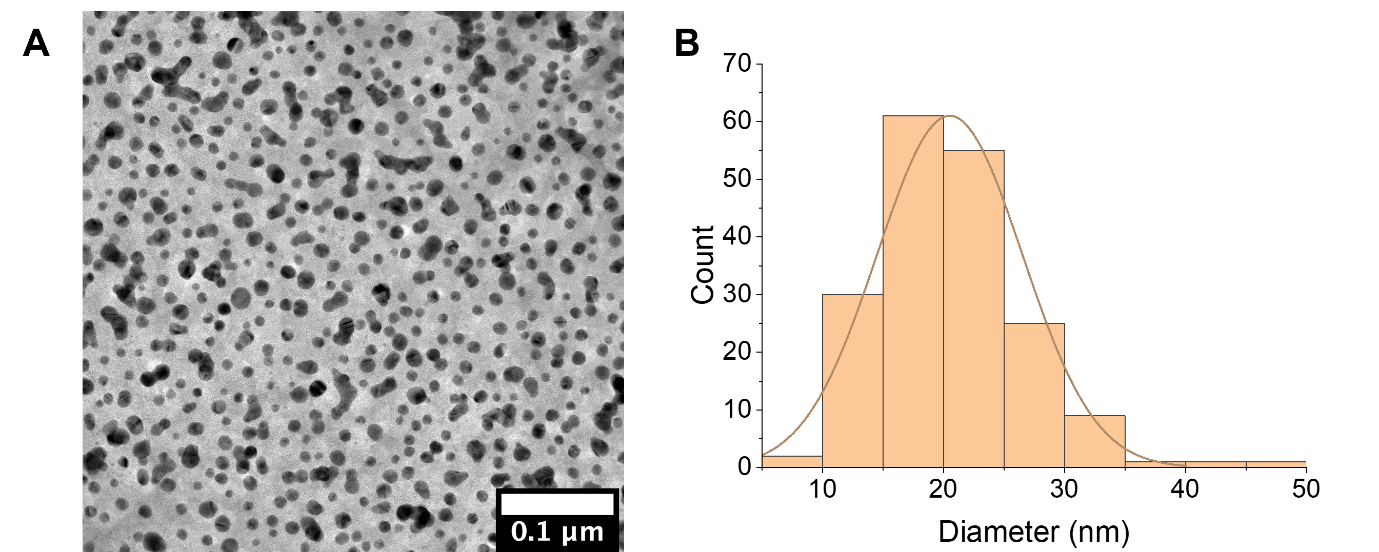


**Supplementary Figure 1:** TEM analysis of AuNPs. A) Representative TEM image B) Histograms from analysis of >150 particles. Average Diameter 19.6 nm.


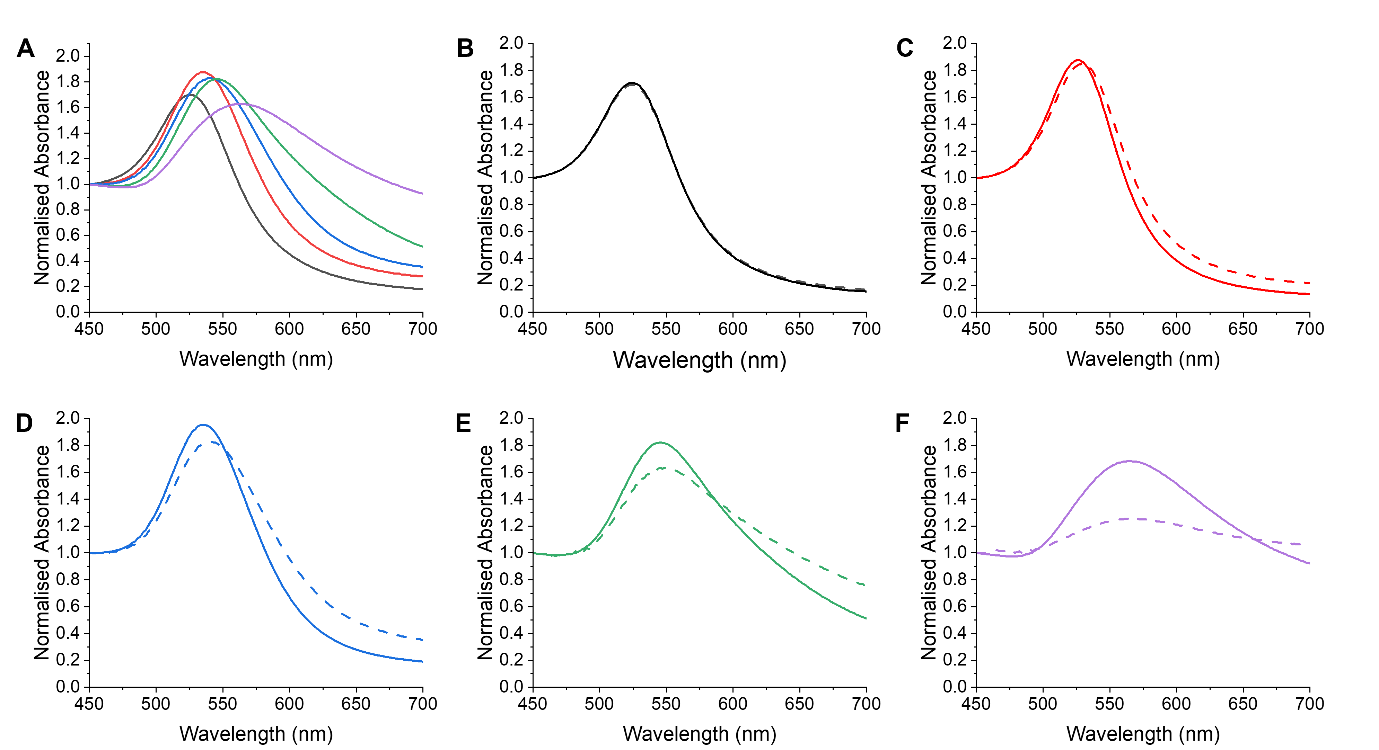


**Supplementary Figure 2**: A) UV-Vis spectra of AuNPs of different sizes where; black = 16 nm, red = 32 nm, blue = 48 nm, green = 76 nm, and purple = 82 nm as determined by wavelength of SPR peak[30]. B) – F) Stability of AuNPs in PBS (dotted line) compared to water (solid line). Particle showed some instability with the larger particles being unstable in PBS, therefore smallest (16 nm) AuNPs were taken further in this study.


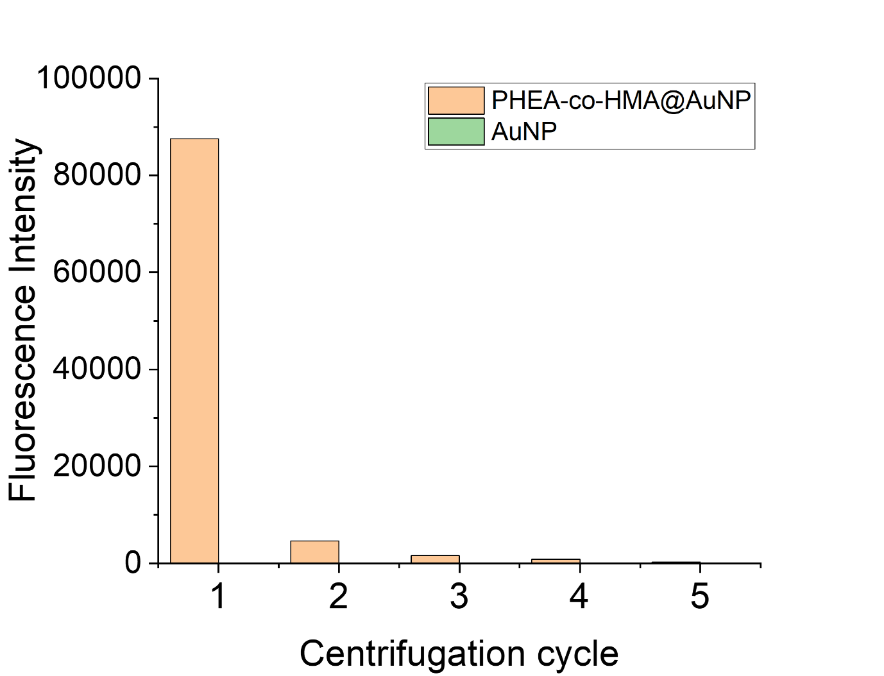


**Supplementary Figure 3**: Fluorescence intensity of supernatant of PHEA-co-Hostasol@AuNP after each centrifugation cycle. Excitation 461 nm, Emission 528 nm.


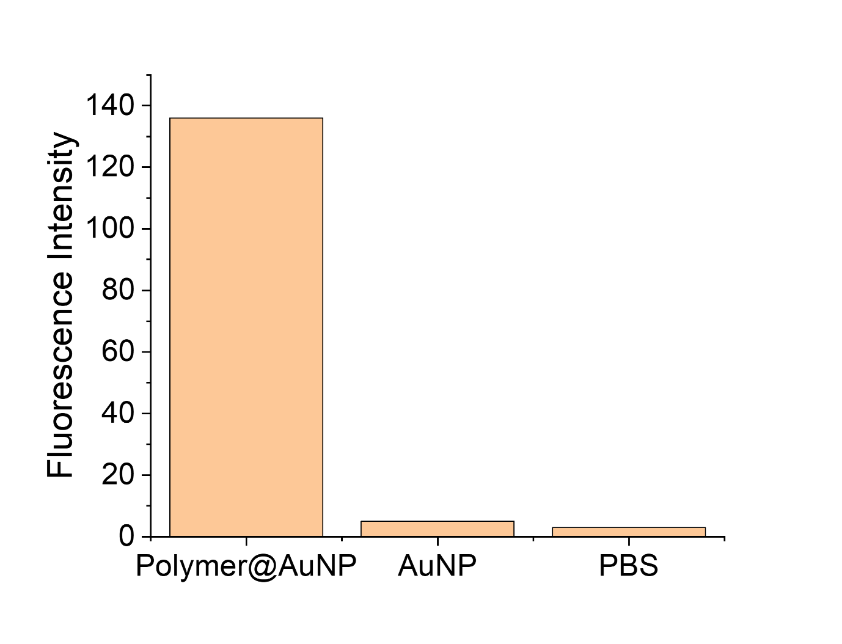


**Supplementary Figure 4**: Fluorescence intensity of PHEA-co-hostasol@AuNP (polymer@AuNP) compared to naked AuNPs and PBS alone. After five cycles we were confident that all excess polymer was removed so any fluorescence associated was due to hostasol adhered to AuNPs. As expected no fluorescence was observable for particles with no fluorescent polymer added.


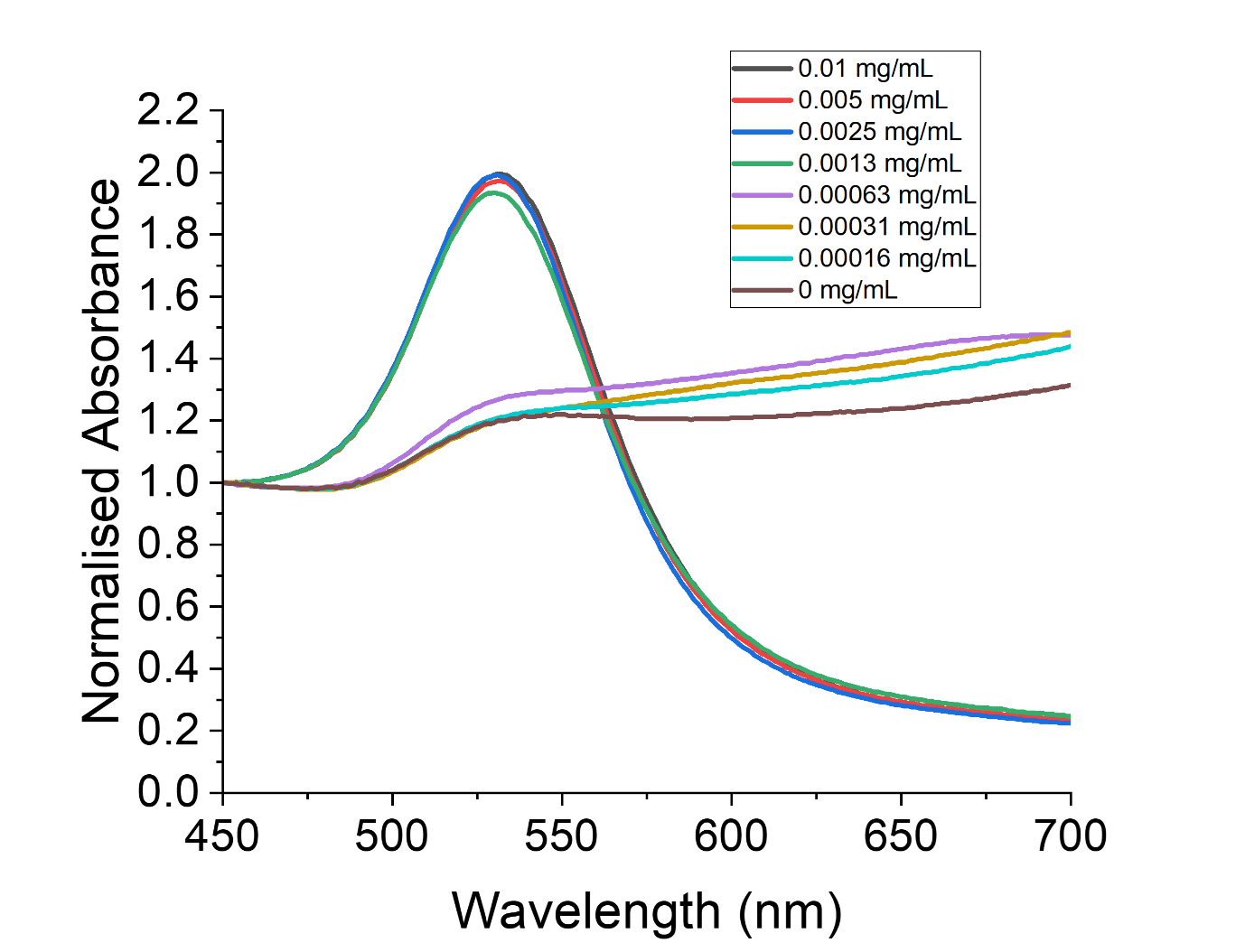


**Supplementary Figure 5:** Amount of polymer required to lead to stable conjugates. Differing amounts of polymer were added to determine concentration required to stabilize from 0.1 mg.mL^-1^. A minimum 0.01 mg.mL^-1^is required to stabilize the AuNPs. 0.1 mg.mL^-1^ was used going forward.


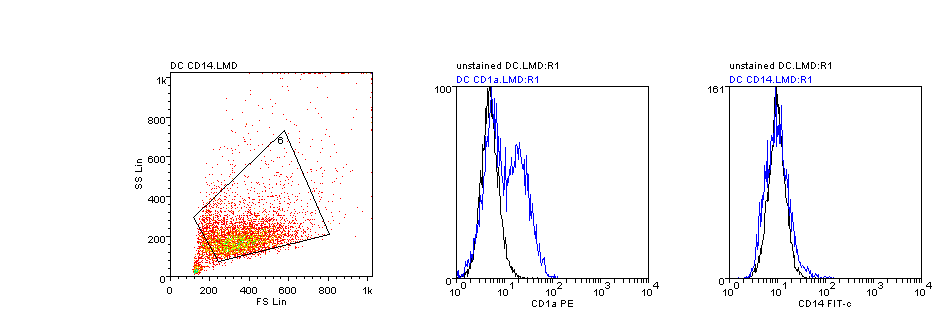


**Supplementary Figure 6:** Monocyte toDCsdifferentiation was assessed using flowcytometry to quantify CD1a, and CD14 expression levels. This data shows absence of CD14 and presence of CD1a indicating monocyte differentiation into DCs.


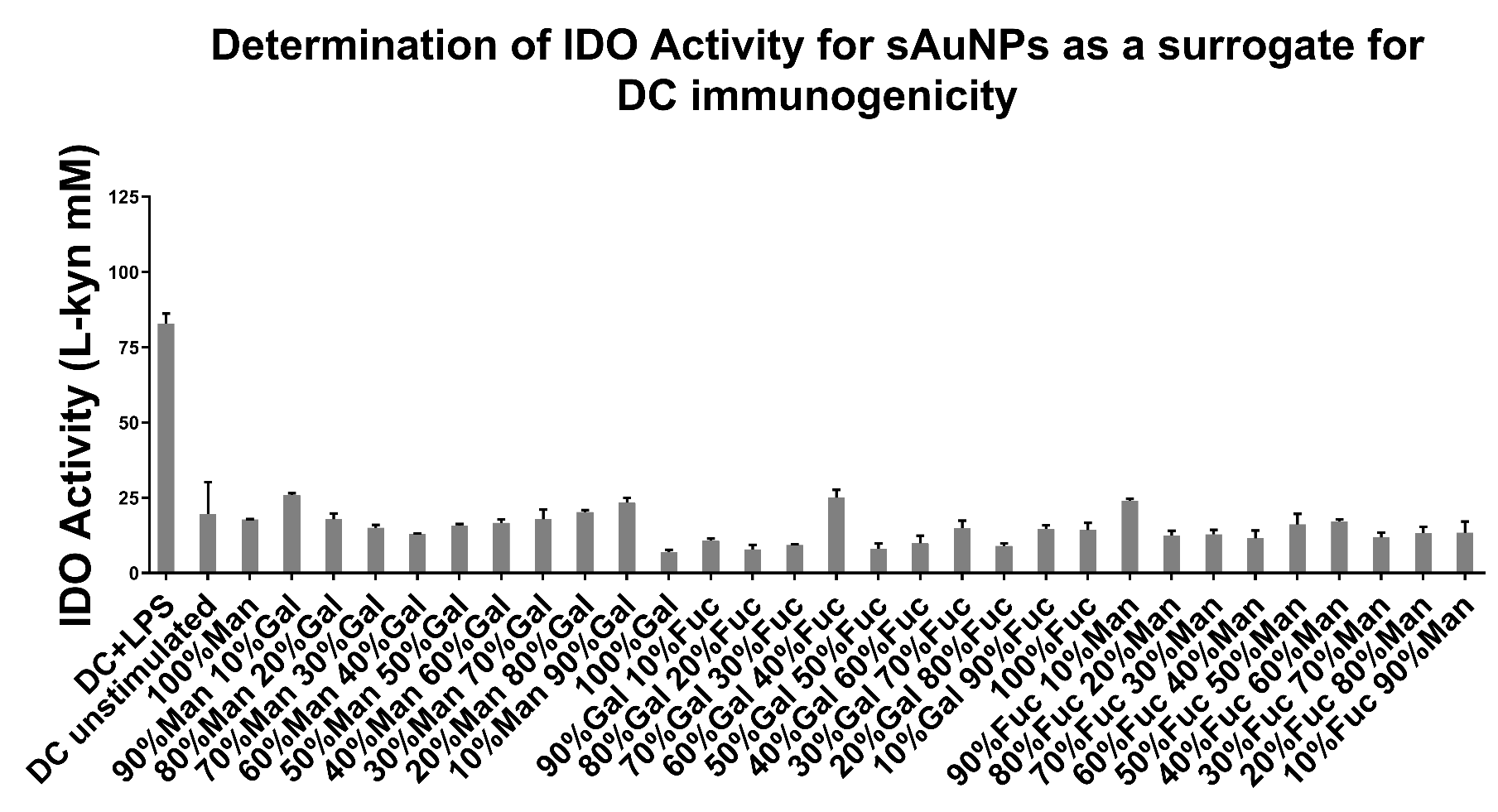


**Supplementary Figure 7:** Estimation of IDO activity using kynurenine as a surrogate for DC immunogenicity after conditioning with sAuNPs. Data show no remarkable increase in IDO activity compared to unstimulated control. This suggests that sAuNPs did not have a stimulatory effect on DCs. Data shown are a mean +-SD of 3 separate donors (n=3).


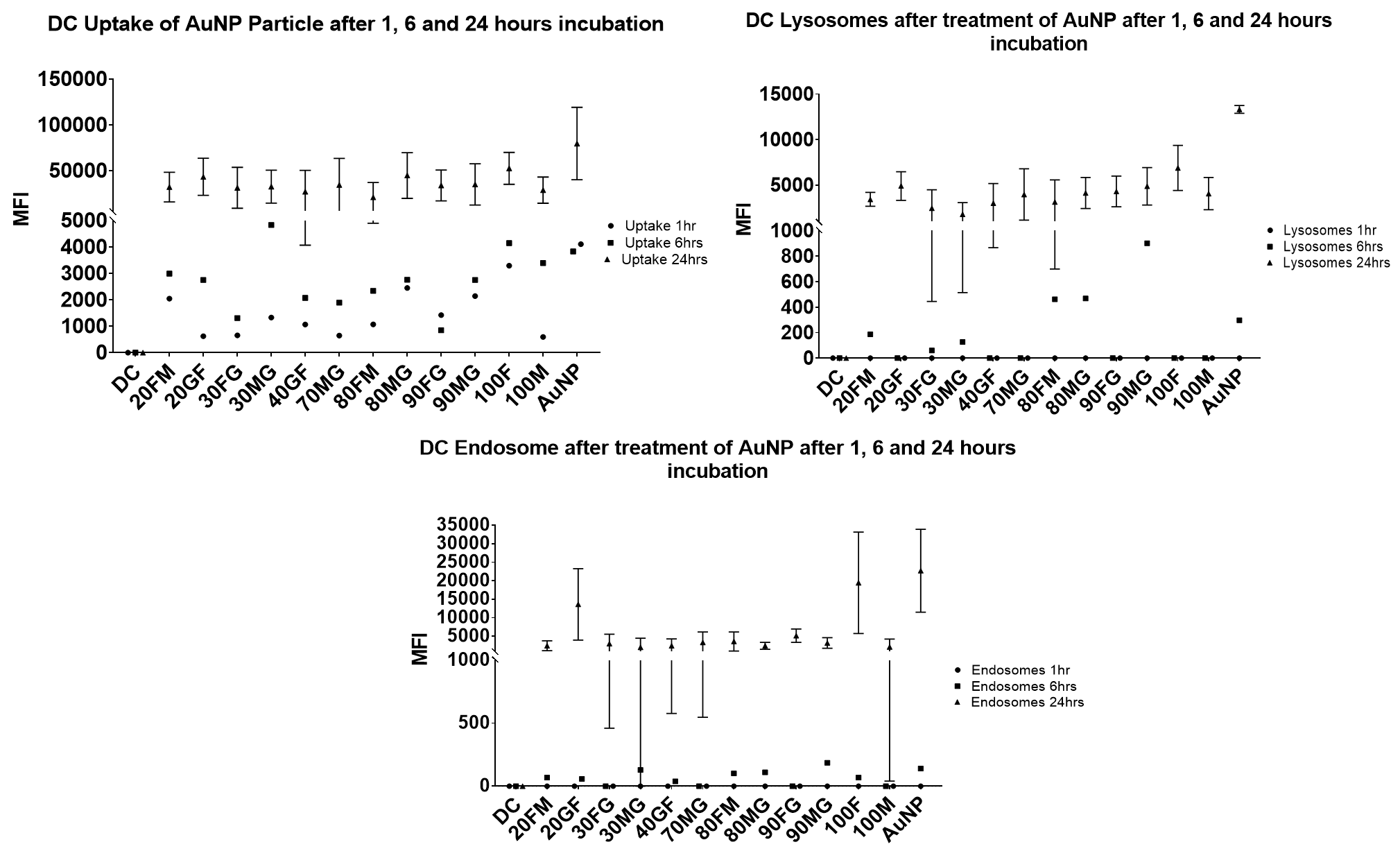


**Supplementary Figure 8:**Time point experiments for sAuNP conditioned DC for the assessment of uptake, lysosomal and endosomal content. Data show similar uptake patterns for conditions in each time points. Increase in lysosomal and endosomal contents can be seen after 24 hours of culture. Data shown as mean ± SD for a single experiment for 1 and 6 hours time points and for >3 donors for 24 hours


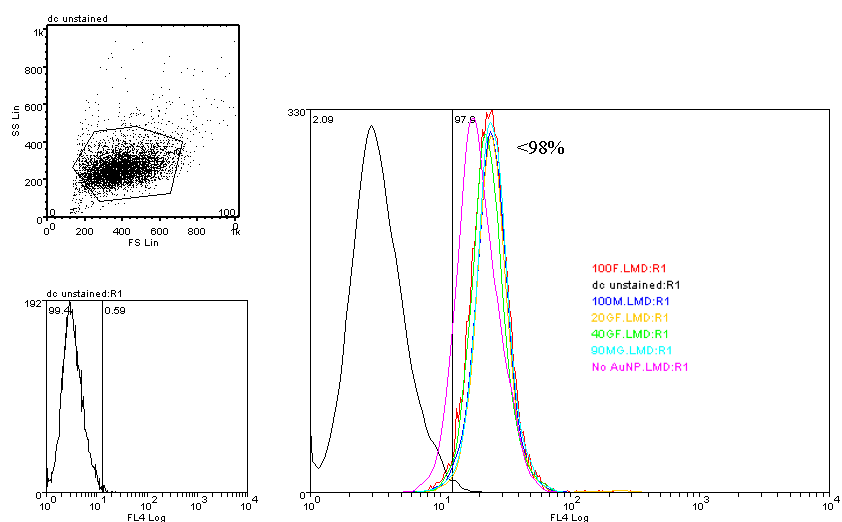


**Supplementary Figure 9:**DCsviability following sAuNP treatment was assessed using Live/Dead assay and flowcytometry. There is no significant difference in DC viability between No AuNP (DC only) and sAuNP treated DCs. Data are representative of 3 independent experiments.

|  |  | 20FM | 20GF | 30MG | 80MG | 90FG | 90MG | 100F | 100M | AuNP |
| --- | --- | --- | --- | --- | --- | --- | --- | --- | --- | --- |
| Co-localization  Lysosome | L-D1 | 139.0625 | 184.1146 | 170.5729 | 142.1875 | 165.1042 | 142.7083 | 179.9479 | 110.1563 | 100 |
|  | L-D2 | 150.9852 | 157.6355 | 157.3892 | 135.2217 | 114.2857 | 115.0246 | 175.6158 | 112.069 | 100 |
|  | L-D3 | 174.6512 | 205.1163 | 173.9535 | 184.8837 | 193.2558 | 176.2791 | 200.4651 | 186.0465 | 100 |
| Co-localization  Endosome | E-D1 | 217.6471 | 136.9748 | 129.4118 | 110.9244 | 94.95798 | 152.1008 | 93.27731 | 168.0672 | 100 |
|  | E-D2 | 241.7417 | 222.2222 | 146.2462 | 195.1952 | 107.2072 | 166.6667 | 135.1351 | 147.1471 | 100 |
|  | E-D3 | 114.5833 | 120.4167 | 118.3333 | 121.0417 | 119.8333 | 108.3333 | 99.16667 | 143.75 | 100 |

**Supplementary Table1:** The raw data from all samples used to quantify the co-localization. L=lysosome; E=endosome; D= donor
